# 3-Nitro-5-(4-pyridinio)benzoate

**DOI:** 10.1107/S160053681002800X

**Published:** 2010-07-24

**Authors:** Xiao-Jun Zhao, Cheng-Jun Hao

**Affiliations:** a College of Chemistry and Chemical Engineering, Pingdingshan University, Pingdingshan 467000, People’s Republic of China

## Abstract

The title compound, C_12_H_8_N_2_O_4_, crystallizes as a zwitterion in which the pyridyl N atom is protonated. The dihedral angle between the benzene and pyridinium rings is 27.9 (2)°. In the crystal, N—H⋯O hydrogen bonds link adjacent zwitterions into a three-dimensional structure.

## Related literature

The title compound was reacted with MgCl_2_ under hydrothermal conditions in an attempt to obtain a new coordination polymer as part of our investigation of pyridine caboxylate coordination polymers. For the advantages of hydro­thermal synthesis, see: Feng *et al.* (2001 ▶); Tao *et al.* (2001 ▶). For the crystal structures of coord­in­ation polymers involving 4-pyridine­carboxyl­ate ligands, see: Lu *et al.* (2003 ▶).
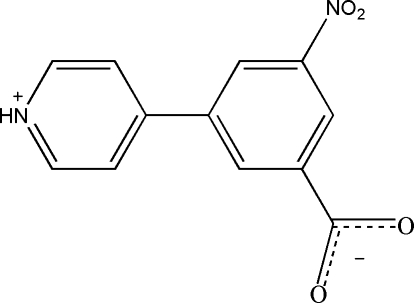


## Experimental

### 

#### Crystal data

C_12_H_8_N_2_O_4_
*M* *_r_* = 244.20
Orthorhombic, 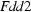

*a* = 16.1215 (14) Å
*b* = 37.126 (3) Å
*c* = 7.1317 (8) Å
*V* = 4268.5 (7) Å^3^
*Z* = 16
Mo *K*α radiation
μ = 0.12 mm^−1^
*T* = 298 K
0.46 × 0.17 × 0.09 mm

#### Data collection

Bruker SMART 1000 CCD area-detector diffractometer
Absorption correction: multi-scan (*SADABS*; Bruker, 2007 ▶) *T*_min_ = 0.948, *T*_max_ = 0.990
4377 measured reflections
1023 independent reflections
621 reflections with *I* > 2σ(*I*)
*R*_int_ = 0.110

#### Refinement

*R*[*F* ^2^ > 2σ(*F* ^2^)] = 0.048
*wR*(*F* ^2^) = 0.118
*S* = 1.02
1023 reflections
163 parameters
1 restraint
H-atom parameters constrained
Δρ_max_ = 0.18 e Å^−3^
Δρ_min_ = −0.22 e Å^−3^

### 

Data collection: *SMART* (Bruker, 2007 ▶); cell refinement: *SAINT* (Bruker, 2007 ▶); data reduction: *SAINT*; program(s) used to solve structure: *SHELXS97* (Sheldrick, 2008 ▶); program(s) used to refine structure: *SHELXL97* (Sheldrick, 2008 ▶); molecular graphics: *SHELXTL* (Sheldrick, 2008 ▶); software used to prepare material for publication: *SHELXL97*.

## Supplementary Material

Crystal structure: contains datablocks I, global. DOI: 10.1107/S160053681002800X/wn2400sup1.cif

Structure factors: contains datablocks I. DOI: 10.1107/S160053681002800X/wn2400Isup2.hkl

Additional supplementary materials:  crystallographic information; 3D view; checkCIF report

## Figures and Tables

**Table 1:** Hydrogen-bond geometry (Å, °)

*D*—H⋯*A*	*D*—H	H⋯*A*	*D*⋯*A*	*D*—H⋯*A*
N2—H2⋯O1^i^	0.86	1.74	2.592 (5)	172

Symmetry code: (i) 
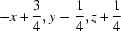
.

**Fig. 1.**
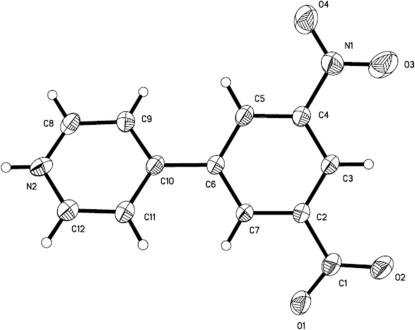
The structure of the title compound, showing the atomic numbering scheme. Non-H atoms are shown with 30% probability displacement ellipsoids. H atoms are drawn as spheres of arbitrary radii.

**Fig. 2.**
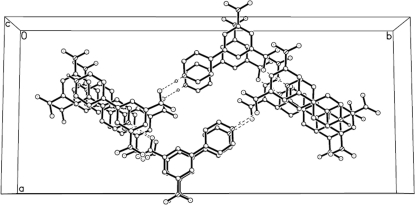
View of the three-dimensional network constructed by N—H···O hydrogen bonding interactions (dashed lines). H atoms not involved in the hydrogen bonds are omitted for clarity.

## supplementary crystallographic information

### Comment

Hydrothermal synthesis has been successful in the preparation of new materials, because problems associated with ligand solubility were minimized and the reactivity of reactants was enhanced during the crystallization process in a heated sealed solution above ambient temperature and pressure (Feng *et al.*, 2001; Tao *et al.*, 2001). Thus, we have reacted 5-(4-pyridyl)-3-nitrobenzoic acid with MgCl_2_ under hydrothermal conditions in an effort to obtain a new coordination polymer as part of further investigation of pyridine caboxylate coordination polymers (Lu *et al.*, 2003). In fact, no complex was formed, but we report here the crystal structure of the starting organic compound.

In the title compound, C_12_H_8_N_2_O_4_, the pyridyl N atom is protonated, and the compound is formally a zwitterion. The carboxyl group and the nitro group are approximately coplanar with the aromatic ring (Fig. 1), as indicated by the O2—C1—C2—C3  and O1—C1—C2—C3 torsion angles of 11.1 (8) ° and -170.2 (5) °, respectively; the O3—N1—C4—C3 and O4—N1—C4—C3 torsion angles are 1.0 (7) and -176.9 (5) °, respectively. Furthermore, the dihedral angle between the benzene ring and the pyridine ring is 27.9 (2) °. In the crystal packing, N—H···O hydrogen bonds stabilize the three-dimensional structure.

### Experimental

A mixture of MgCl_2_ (0.1 mmol, 0.01g), 5-(4-pyridyl)-3-nitrobenzoic acid (0.1 mmol, 0.025 g) and 10 ml of H_2_O was loaded in a 20 ml Teflon-lined stainless steel vessel and heated at 303K for 3 days. Colourless crystals were obtained when the solution was slowly cooled to room temperature.

### Refinement

H atoms were placed at calculated positions and were treated as riding on the parent C or N atoms with C—H = 0.93 Å, N—H = 0.86 Å, and with *U*_iso_(H) = 1.2 *U*_eq_(C, N). In the absence of significant anomalous scattering Friedel pairs were merged.

### Crystal data

**Table:**

C_12_H_8_N_2_O_4_	*F*(000) = 2016
*M**_r_* = 244.20	*D*_x_ = 1.520 Mg m^−^^3^
Orthorhombic, *F**d**d*2	Mo *K*α radiation, λ = 0.71073 Å
Hall symbol: F 2 -2d	Cell parameters from 638 reflections
*a* = 16.1215 (14) Å	θ = 2.8–26.3°
*b* = 37.126 (3) Å	µ = 0.12 mm^−^^1^
*c* = 7.1317 (8) Å	*T* = 298 K
*V* = 4268.5 (7) Å^3^	Needle, colourless
*Z* = 16	0.46 × 0.17 × 0.09 mm

### Data collection

**Table:**

Bruker SMART 1000 CCD area-detector diffractometer	1023 independent reflections
Radiation source: fine-focus sealed tube	621 reflections with *I* > 2σ(*I*)
graphite	*R*_int_ = 0.110
φ and ω scans	θ_max_ = 25.0°, θ_min_ = 2.2°
Absorption correction: multi-scan (*SADABS*; Bruker, 2007)	*h* = −19→11
*T*_min_ = 0.948, *T*_max_ = 0.990	*k* = −43→43
4377 measured reflections	*l* = −8→8

### Refinement

**Table:**

Refinement on *F*^2^	Primary atom site location: structure-invariant direct methods
Least-squares matrix: full	Secondary atom site location: difference Fourier map
*R*[*F*^2^ > 2σ(*F*^2^)] = 0.048	Hydrogen site location: inferred from neighbouring sites
*wR*(*F*^2^) = 0.118	H-atom parameters constrained
*S* = 1.02	*w* = 1/[σ^2^(*F*_o_^2^) + (0.0406*P*)^2^ + 0.170*P*] where *P* = (*F*_o_^2^ + 2*F*_c_^2^)/3
1023 reflections	(Δ/σ)_max_ < 0.001
163 parameters	Δρ_max_ = 0.18 e Å^−^^3^
1 restraint	Δρ_min_ = −0.22 e Å^−^^3^

### Special details

**Table:**

Geometry. All esds (except the esd in the dihedral angle between two l.s. planes) are estimated using the full covariance matrix. The cell esds are taken into account individually in the estimation of esds in distances, angles and torsion angles; correlations between esds in cell parameters are only used when they are defined by crystal symmetry. An approximate (isotropic) treatment of cell esds is used for estimating esds involving l.s. planes.
Refinement. Refinement of F^2^ against ALL reflections. The weighted R-factor wR and goodness of fit S are based on F^2^, conventional R-factors R are based on F, with F set to zero for negative F^2^. The threshold expression of F^2^ > 2sigma(F^2^) is used only for calculating R-factors(gt) etc. and is not relevant to the choice of reflections for refinement. R-factors based on F^2^ are statistically about twice as large as those based on F, and R- factors based on ALL data will be even larger.

### Fractional atomic coordinates and isotropic or equivalent isotropic displacement parameters (Å^2^)

**Table:**

	*x*	*y*	*z*	*U*_iso_*/*U*_eq_	
N1	−0.0034 (3)	0.07102 (12)	0.9143 (9)	0.0630 (15)	
N2	0.3542 (3)	−0.05673 (10)	0.8083 (7)	0.0543 (14)	
H2	0.3836	−0.0760	0.8109	0.065*	
O1	0.3097 (2)	0.13480 (9)	0.5977 (7)	0.0701 (13)	
O2	0.2043 (2)	0.16759 (9)	0.7037 (7)	0.0731 (14)	
O3	−0.0420 (2)	0.09929 (10)	0.9147 (9)	0.0919 (17)	
O4	−0.0331 (2)	0.04219 (10)	0.9681 (8)	0.0856 (17)	
C1	0.2386 (4)	0.13874 (13)	0.6766 (9)	0.0526 (15)	
C2	0.2001 (3)	0.10398 (12)	0.7393 (8)	0.0435 (14)	
C3	0.1187 (3)	0.10352 (12)	0.7997 (8)	0.0486 (15)	
H3	0.0881	0.1247	0.8046	0.058*	
C4	0.0835 (3)	0.07138 (12)	0.8524 (9)	0.0474 (15)	
C5	0.1277 (3)	0.03937 (12)	0.8486 (8)	0.0451 (14)	
H5	0.1020	0.0179	0.8822	0.054*	
C6	0.2104 (3)	0.03957 (11)	0.7944 (7)	0.0403 (12)	
C7	0.2461 (3)	0.07216 (11)	0.7400 (8)	0.0428 (14)	
H7	0.3015	0.0727	0.7035	0.051*	
C8	0.2731 (3)	−0.05830 (13)	0.7825 (8)	0.0508 (15)	
H8	0.2480	−0.0807	0.7685	0.061*	
C9	0.2244 (3)	−0.02788 (12)	0.7757 (8)	0.0475 (14)	
H9	0.1675	−0.0300	0.7580	0.057*	
C10	0.2600 (3)	0.00589 (12)	0.7950 (8)	0.0420 (13)	
C11	0.3461 (3)	0.00687 (12)	0.8235 (9)	0.0522 (15)	
H11	0.3734	0.0288	0.8378	0.063*	
C12	0.3890 (3)	−0.02476 (13)	0.8300 (9)	0.0575 (17)	
H12	0.4459	−0.0238	0.8507	0.069*	

### Atomic displacement parameters (Å^2^)

**Table:**

	*U*^11^	*U*^22^	*U*^33^	*U*^12^	*U*^13^	*U*^23^
N1	0.044 (3)	0.056 (3)	0.089 (4)	0.001 (2)	0.002 (3)	−0.004 (3)
N2	0.055 (3)	0.036 (2)	0.072 (4)	0.014 (2)	0.003 (3)	0.005 (3)
O1	0.063 (3)	0.041 (2)	0.106 (4)	−0.0047 (18)	0.024 (3)	0.005 (2)
O2	0.066 (3)	0.0369 (19)	0.116 (4)	0.0041 (18)	0.007 (3)	0.011 (3)
O3	0.063 (2)	0.065 (3)	0.149 (5)	0.020 (2)	0.030 (3)	0.006 (3)
O4	0.056 (2)	0.058 (2)	0.143 (5)	−0.009 (2)	0.019 (3)	0.012 (3)
C1	0.057 (4)	0.035 (3)	0.066 (4)	−0.003 (3)	0.001 (3)	0.011 (3)
C2	0.042 (3)	0.032 (2)	0.057 (4)	−0.001 (2)	−0.001 (3)	0.000 (2)
C3	0.046 (3)	0.030 (3)	0.070 (4)	0.003 (2)	−0.001 (3)	−0.002 (3)
C4	0.041 (3)	0.034 (3)	0.067 (4)	−0.001 (2)	0.000 (3)	0.000 (3)
C5	0.046 (3)	0.031 (3)	0.058 (4)	0.001 (2)	0.001 (3)	0.003 (3)
C6	0.040 (3)	0.031 (3)	0.049 (3)	−0.002 (2)	−0.001 (3)	0.000 (3)
C7	0.035 (3)	0.031 (2)	0.061 (4)	0.001 (2)	0.000 (3)	−0.001 (3)
C8	0.057 (4)	0.032 (3)	0.063 (4)	0.001 (2)	−0.003 (3)	0.008 (3)
C9	0.044 (3)	0.038 (3)	0.060 (4)	−0.004 (2)	0.001 (3)	0.001 (3)
C10	0.045 (3)	0.031 (3)	0.051 (4)	0.002 (2)	0.003 (3)	0.003 (2)
C11	0.051 (3)	0.035 (3)	0.070 (4)	−0.001 (2)	0.004 (3)	0.004 (3)
C12	0.049 (3)	0.042 (3)	0.082 (5)	0.005 (3)	0.005 (3)	0.008 (3)

### Geometric parameters (Å, °)

**Table:**

N1—O3	1.220 (5)	C5—C6	1.388 (6)
N1—O4	1.233 (5)	C5—H5	0.9300
N1—C4	1.469 (7)	C6—C7	1.395 (6)
N2—C12	1.322 (6)	C6—C10	1.485 (6)
N2—C8	1.323 (6)	C7—H7	0.9300
N2—H2	0.8600	C8—C9	1.376 (6)
O1—C1	1.284 (6)	C8—H8	0.9300
O2—C1	1.221 (6)	C9—C10	1.386 (6)
C1—C2	1.500 (6)	C9—H9	0.9300
C2—C3	1.381 (7)	C10—C11	1.403 (6)
C2—C7	1.394 (6)	C11—C12	1.364 (6)
C3—C4	1.374 (6)	C11—H11	0.9300
C3—H3	0.9300	C12—H12	0.9300
C4—C5	1.386 (6)		
			
O3—N1—O4	123.2 (5)	C5—C6—C7	118.6 (4)
O3—N1—C4	118.6 (5)	C5—C6—C10	120.8 (4)
O4—N1—C4	118.1 (4)	C7—C6—C10	120.6 (4)
C12—N2—C8	118.4 (4)	C2—C7—C6	121.1 (4)
C12—N2—H2	120.8	C2—C7—H7	119.4
C8—N2—H2	120.8	C6—C7—H7	119.4
O2—C1—O1	125.0 (5)	N2—C8—C9	122.2 (5)
O2—C1—C2	121.3 (5)	N2—C8—H8	118.9
O1—C1—C2	113.7 (4)	C9—C8—H8	118.9
C3—C2—C7	119.6 (4)	C8—C9—C10	120.2 (4)
C3—C2—C1	119.8 (4)	C8—C9—H9	119.9
C7—C2—C1	120.7 (4)	C10—C9—H9	119.9
C4—C3—C2	119.3 (4)	C9—C10—C11	116.6 (4)
C4—C3—H3	120.4	C9—C10—C6	122.6 (4)
C2—C3—H3	120.4	C11—C10—C6	120.8 (4)
C3—C4—C5	121.8 (4)	C12—C11—C10	119.0 (5)
C3—C4—N1	119.0 (4)	C12—C11—H11	120.5
C5—C4—N1	119.2 (5)	C10—C11—H11	120.5
C4—C5—C6	119.6 (4)	N2—C12—C11	123.6 (5)
C4—C5—H5	120.2	N2—C12—H12	118.2
C6—C5—H5	120.2	C11—C12—H12	118.2
			
O2—C1—C2—C3	11.2 (9)	C3—C2—C7—C6	2.3 (9)
O1—C1—C2—C3	−170.0 (5)	C1—C2—C7—C6	−178.7 (5)
O2—C1—C2—C7	−167.8 (6)	C5—C6—C7—C2	−0.1 (8)
O1—C1—C2—C7	10.9 (8)	C10—C6—C7—C2	−179.8 (5)
C7—C2—C3—C4	−2.6 (8)	C12—N2—C8—C9	0.7 (9)
C1—C2—C3—C4	178.4 (5)	N2—C8—C9—C10	0.4 (9)
C2—C3—C4—C5	0.7 (8)	C8—C9—C10—C11	−0.8 (8)
C2—C3—C4—N1	−179.3 (6)	C8—C9—C10—C6	−178.2 (5)
O3—N1—C4—C3	0.9 (8)	C5—C6—C10—C9	25.9 (8)
O4—N1—C4—C3	−177.2 (6)	C7—C6—C10—C9	−154.3 (5)
O3—N1—C4—C5	−179.0 (6)	C5—C6—C10—C11	−151.4 (6)
O4—N1—C4—C5	2.8 (8)	C7—C6—C10—C11	28.4 (8)
C3—C4—C5—C6	1.5 (9)	C9—C10—C11—C12	0.2 (9)
N1—C4—C5—C6	−178.5 (5)	C6—C10—C11—C12	177.6 (5)
C4—C5—C6—C7	−1.8 (8)	C8—N2—C12—C11	−1.3 (10)
C4—C5—C6—C10	178.0 (5)	C10—C11—C12—N2	0.9 (10)

### Hydrogen-bond geometry (Å, °)

**Table:**

*D*—H···*A*	*D*—H	H···*A*	*D*···*A*	*D*—H···*A*
N2—H2···O1^i^	0.86	1.74	2.592 (5)	172

Symmetry codes: (i) −*x*+3/4, *y*−1/4, *z*+1/4.
